# Developing, Implementing and Sustaining Community Engaged Research Within an Underserved Community Clinic

**DOI:** 10.1093/geroni/igaf122.857

**Published:** 2025-12-31

**Authors:** Ana-Maria Vranceanu, Kate McDermott, Courtney Kilduff, Clara Vanderheide, Yoojee Kim, Natalia Giraldo-Santiago, Jonathan Greenberg, Christine Ritchie

**Affiliations:** Massachusetts General Hospital, Boston, Massachusetts, United States; Massachusetts General Hospital Center for Health Outcomes and Interdisciplinary Research, Boston, Massachusetts, United States; Massachusetts General Hospital Center for Health Outcomes and Interdisciplinary Research, Boston, Massachusetts, United States; Massachusetts General Hospital Center for Health Outcomes and Interdisciplinary Research, Boston, Massachusetts, United States; Massachusetts General Hospital Center for Health Outcomes and Interdisciplinary Research, Boston, Massachusetts, United States; Massachusetts General Hospital Center for Health Outcomes and Interdisciplinary Research, Boston, Massachusetts, United States; Massachusetts General Hospital Center for Health Outcomes and Interdisciplinary Research, Boston, Massachusetts, United States; Massachusetts General Hospital, Boston, Massachusetts, United States

## Abstract

Chronic musculoskeletal pain (pain > 3-6 months) is prevalent, costly, challenging to treat and a national priority area. Older adults have the highest rates of chronic pain, and these are expected to increase over the next three decades. Mounting evidence suggests that older adults with chronic pain from disadvantaged populations (e.g., lower social and economic resources, racial and ethnic minorities) are at the highest risk, but underserved community clinics where these older adults seek care, lack evidence based nonpharmacological pain treatments tailored to their needs. This presentation will describe the process of co-design, testing and implementation of a mind-body activity program tailored for the unique needs of these older adults with chronic pain, through community engaged methods that included a partnership with primary care doctors, nurses, medical interpreters, leadership and patients within an underserved community clinic. We will specifically focus on: 1) strategies used to establish a successful partnership (e.g., identifying key stakeholders, building trust, and shared decision-making); 2) conducting community needs assessments (e.g., listening sessions, surveys and focus groups); 3) utilizing cultural and linguistic adaptations; 4) integrating research into the clinical workflow (e.g., minimizing burden, flexible scheduling); 5) leveraging clinic providers (e.g., nurses, social workers, psychologists); 6) ongoing feedback from stakeholders for optimization of procedures; 7) co-creating dissemination strategies (accessible results sharing at multiple time-points, celebrating milestones); 8) ensuring mutual benefit (capacity building, resource sharing); 9) maintaining long term relationships (sustainability planning through attention to intervention cost, billing and patient co-pays) and 10) ethical considerations (transparency and reciprocity).

